# Socioeconomic inequities in specialized health services use following COVID-19 in individuals from Southern Brazil

**DOI:** 10.1186/s12913-023-09476-7

**Published:** 2023-05-25

**Authors:** Yohana Pereira Vieira, Juliana Quadros Santos Rocha, Rinelly Pazinato Dutra, Lorrany da Silva Nunes, Suele Manjourany Silva Duro, Mirelle de Oliveira Saes

**Affiliations:** 1 Postgraduate Programme in Health sciences, Federal University of Rio Grande, Rio Grande, Rio Grande do Sul Brazil; 2 Postgraduate Programme in Public Health, Federal University of Rio Grande, Rio Grande, Rio Grande do Sul Brazil; 3 Postgraduate Programme in Nursing, Federal University of Pelotas, Pelotas, Brazil

**Keywords:** Health Services, Health Status Disparities, Health Inequality Monitoring, COVID-19, Brazil, Access

## Abstract

**Background:**

Evidence on inequalities in the health services use is important for public policy formulation, even more so in a pandemic context. The aim of this study was to evaluate socioeconomic inequities in the specialized health use services according to health insurance and income, following COVID-19 in individuals residing in Southern Brazil.

**Methods:**

This was a cross-sectional telephone survey with individuals aged 18 years or older diagnosed with symptomatic COVID-19 using the RT-PCR test between December 2020 and March 2021. Questions were asked about attendance at a health care facility following COVID-19, the facilities used, health insurance and income. Inequalities were assessed by the following measures: Slope Index of Inequality (SII) and Concentration Index (CIX). Adjusted analyses were performed using Poisson regression with robust variance adjustment using the Stata 16.1 statistical package.

**Results:**

2,919 people (76.4% of those eligible) were interviewed. Of these, 24.7% (95%CI 23.2; 36.3) used at least one specialized health service and 20.3% (95%CI 18.9; 21.8) had at least one consultation with specialist doctors after diagnosis of COVID-19. Individuals with health insurance were more likely to use specialized services. The probability of using specialized services was up to three times higher among the richest compared to the poorest.

**Conclusions:**

There are socioeconomic inequalities in the specialized services use by individuals following COVID-19 in the far south of Brazil. It is necessary to reduce the difficulty in accessing and using specialized services and to extrapolate the logic that purchasing power transposes health needs. The strengthening of the public health system is essential to guarantee the population’s right to health.

## Introduction

Health services use represents the core of the functioning of health systems, being an indirect way to verify access to and equity in these services [1]. Numerous conditions are associated with the health services use, so that geographical accessibility and sociocultural and economic factors, for example, play a primary role in the demand for them [2].

The brazilian health model is based on the Unified Health System (SUS), which is structured through the sharing of responsibilities between the three spheres of the federation – Union, States and Municipalities [3]. SUS is a universal State policy that expanded the Brazilian social protection system [4] assuming that health is a right for all and a duty of the State [5] which, in turn, instead, it must offer comprehensive and articulated services at different levels of care – primary health care in public services, medical services (secondary and tertiary care) and specialized services. SUS promotes universal, equitable and decentralized access to users [6–9].

The health services use is defined as public-private due to the possibility of user access not only via the SUS, but also via private health services and via a health plan, since the SUS is not only public but is also composed of a vast network of contracted private services that are remunerated with fiscal resources aimed at health and their participation in the SUS is complementary [10]. However, the restricted supply specialized services use in Brazil makes it difficult to guarantee comprehensiveness in the SUS, due to the difficulty of access in circumstances that allow adequate and timely use and the recurrent public dependence on agreements with the private sector [11]. The specialized services use becomes more critical in small cities, given the great distances to urban areas, insufficient and inadequate number of professionals and unavailability and/or high costs associated with health transportation [12–14].

Although, historically, there are bottlenecks in specialized services access in Brazil, especially by the most vulnerable populations, the evolution of the health care network over the years shows that the number of clinics and outpatient clinics that offer specialized services has grown, whether in public or private establishments. However, it should be noted that the availability of specialized services in clinics occurs mostly in the private sector [15]. During the COVID-19 pandemic scenario, specialized services acess was hampered due to insufficient availability of services and health professionals, as well as care devices being concentrated in the private sector. This situation, which exacerbates fragmentation of care, highlights existing disparities and weakens the guarantee of access to health care by the population [16, 17]. Also noteworthy is the eminent increase in demand for specialized services due to the consequences of COVID-19, the so-called Post-COVID-19 Syndrome, which affects approximately 50% of those infected and can last for more than a year [18].

Brazil has expressive social and economic inequities, making it one of the most unequal countries in the Americas [15, 19]. Studies show that access to health services in general is precarious for a large part of the population, especially people with low education and income, and without access to private health insurance [20, 21]. It is precisely in this scenario of socioeconomic and health inequalities that the COVID-19 pandemic is occurring. It can be seen that more marginalized groups and those in situations of social vulnerability are subject to the worst outcomes of the disease [22]. Health inequalities are negatively related to the pandemic scenario in two ways, because both inequalities have been accentuated during this period, and the course of the pandemic has been severely affected by the disparities evidenced. Moreover, in both cases the population in situations of socioeconomic vulnerability has been the most affected [23, 24].

In this sense, it is of utmost importance that evidence on health inequalities be presented and taken into consideration to underpin and support the formulation and evaluation of public policies [19], even more so in a complex and adverse context such as the COVID-19 pandemic. With regard to access to specialized health services in this scenario, we found a scarcity of studies that seek to evaluate such aspects, which demonstrates the relevance of this research and its originality. Therefore, the objective of the present study was to evaluate the socioeconomic inequities in the specialized health services use according to health insurance and income, following COVID-19 in individuals residing in Southern Brazil.

## Methods

This was a cross-sectional telephone survey carried out in the city of Rio Grande in the far south of the state of Rio Grande do Sul. Rio Grande is a port city, covering 2,817 km², with a population of 212,881 inhabitants [25]. The municipality has a Human Development Index of 0.744 and a Gini index of 0.52 (IBGE, 2023). It has 10,000 beneficiaries of Brazil aid and has a coverage of 64.1% of primary health care and a per capita gross domestic product (GDP) of R$47,045.23” [25, 26]. The study protocol was approved by the Health Research Ethics Committee of the Federal University of Rio Grande (Certificate of Submission for Ethical Appraisal No. 39081120.0.0000.5324). All participants verbally agreed to participate in the study.

The sample included individuals aged 18 years or older residing in Rio Grande who had COVID-19 symptoms and were diagnosed as having COVID-19 by RT-PCR testing between December 2020 and March 2021 and who underwent treatment in the same city. We excluded individuals with functional limitations and/or advanced neurological diseases that made it impossible to answer the questionnaire, as well as those who were in long-stay institutions or deprived of liberty (prisons). Individuals, who were not located after five attempts, i.e. once by telephone, once by whatsapp and three home visits, were considered to be losses.

We contacted the epidemiological health surveillance service of the municipality of Rio Grande to identify adults and elderly individuals infected with COVID-19 in the period investigated, creating a list of individuals with positive RT-PCR results and their respective data (name, address, telephone number and presence of symptoms). Using the list of individuals eligible for the study, we started data collection by telephone interviews. Data collection was carried out by previously trained interviewers, who went through a selection process and later underwent training that lasted for a total of 24 h. In addition to the telephone interviews, home visits were offered for face-to-face data collection, if required.

Data collection occurred from July to October 2021. The answers to the questionnaires were collected electronically (tablet) using the REDcap program and smartphones for telephone calls. The questionnaire was tested and its understanding was verified through a pilot study with telephone and face-to-face interview with individuals from other municipalities who tested positive for COVID-19 in the period prior to the survey data collection. The questionnaire sections on sociodemographic questions, COVID-19  symptomatology, and health service use were constructed by the researchers and tested via telephone and face-to-face interview in the pilot study. Other parts of the questionnaire were obtained from scales already validated in the literature. Calls were recorded to ensure the safety of the researcher and the interviewee, using a free cell phone application (Callmaster), stored in an email account. The questionnaire took approximately 15 min to answer.

Participants were asked whether they had attended any health services after COVID-19. The following services were listed: Use of urgency and emergency services (public emergency room, public urgent care unit, private emergency care room, private urgent care unit), neurologist, pulmonologist, cardiologist, psychiatrist, psychologist, physiotherapist and speech therapist. Two summary variables were created: specialist doctors (pulmonologist, cardiologist, psychiatrist and neurologist) and specialized services (neurologist, pulmonologist, cardiologist, psychiatrist, psychologist, physiotherapist, and speech therapist).

The urgent care use outcome and the emergency health service use outcome were investigated based on the affirmative answer to the following question: “Following COVID-19, how many times did you need to be seen at…” and the sites evaluated were public emergency room, public urgent care unit, private emergency room and private urgent care unit. Regarding specialized services, the interviewees were asked the following question: “Following COVID-19, did you need to seek specialized care with (you can check as many options as you want): pulmonologist, neurologist, cardiologist, psychiatrist, psychologist, physiotherapist, speech therapist. The outcomes were arrived at by analysis of the dichotomous answers (yes/no).

The answer to the health insurance variable was dichotomous, i.e. “yes” or “no”. Income in BRL was categorized into groups: US$ 0-197, 197.35–394.30, 395-788.60, and 788.80 or more. The following covariates were used as adjustment for possible confounding: sex (female/male), age group (18–59/ 60 years or more), skin color (white, yellow/black or brown), education (never studied/ elementary education / high school education / higher education).

Inequality in the specialized health services use according to health insurance and income were estimated using complex measures of inequality, such as the Slope Index of Inequality (SII) for absolute inequality and Concentration Index (CIX) for relative inequality.

Absolute inequality is like the difference in occurrence measures (prevalence, incidence, mortality) between groups, i.e. by subtracting extreme values, and is often expressed in percentage points (p.p.). The Slope Index of Inequality (SII), or absolute inequality index, is another measure of absolute inequality, used specifically for stratification variables that are ordinal (generally indicators of indicators such as income groups, wealth indexes or education). The SII is calculated as the difference, in percentage points, between the estimated values for the extreme groups of the stratification variable. Although the SII was designed based on linear regression, in general, logistic regression is more appropriate when it comes to health coverage or prevalence indicators, as it avoids linear predictions outside the expected range for proportions (from 0 to 100). When it comes to a proportion, both the absolute difference between the groups’ estimates and the SII range from − 100 to 100 p.p., and values close to zero are expected in the absence of inequality. Positive values reflect that the health indicator of interest, whether coverage of an intervention or prevalence of a health problem, is more frequent in the most favored group - for example, in the richest or most educated group. This is considered an inequality in greater convergence for the richest. Negative values suggest that the health indicator is more prevalent in the most disadvantaged group - for example, the poorest or least educated group - which configures inequalities in convergence with the poorest.

The SII represents the absolute difference, in predicted values, of a health indicator between the most advantaged and least advantaged individuals in terms of socioeconomic indicators, taking into account the entire stratifier distribution through a regression model. The IBS is calculated as the difference, in percentage points (p.p.), between the estimated values for the extreme groups of the stratification variable ranging from − 100 to 100 p.p., and values close to zero are expected in the absence of inequality [27]. Relative measures have the potential to highlight how uneven estimates are across groups. It can be calculated, for example, by dividing the values (of prevalence, coverage, etc.) corresponding to the richest group by the value of the poorest group. It informs the excess percentage of a category in relation to the other, or how many times higher is the prevalence in a group compared to another. The Concentration Index (CIX), or concentration index, which, like the SII, takes into account all categories of the stratification variable is an analogue of the Gini index - it ranges from − 1 to + 1, assumes zero as equality, and the further away from zero the values are, the greater the relative inequality. The CIX value corresponds to twice the area between a diagonal line that would represent perfect equality between the groups and the curve that expresses the observed coverage for each cumulative percentage of the study population. When coverage is greater among the richest, the generated area is below the diagonal line, and the opposite is evidenced when coverage is greater among the poorest. As with absolute measures, when the coverage of an intervention is evaluated in relation to wealth subgroups, positive values indicate differences in greater convergence for the richest while negative values mean differences in convergence for the poorest. Some studies, such as ours, also present the CIX as values multiplied by 100, for reasons of data visualization, together with measures of absolute inequalities, without changing its interpretation. The CIX is analogous to the Gini index - it ranges from − 1 to + 1, assumes zero to be equality, and the further away from zero the values are, the greater the relative inequality. Thus, positive values indicate pro-rich differences and negative values mean pro-poor differences. Some studies, like ours, also present the CIX as values multiplied by 100, for data visualization reasons, next to absolute inequality measures, without this changing their interpretation [27].

Descriptive data are presented as proportions and 95% confidence intervals (95%CI). We used Poisson regression to assess the relationship between health services use following COVID-19 according to income and health insurance plan. Adjusted analyses were performed using Poisson regression with robust variance adjustment. All associations with 95%CI without overlap between categories were considered statistically significant. Data were analyzed using the Stata 16.1 statistical package.

## Results

A total of 3,822 participants testing positive for COVID-19 were eligible for the study; after losses (631) and refusals (272), 2,919 (76.4% of those eligible) were interviewed. Of these, 24.7% (95%CI 23.2;36.3) used at least one specialist health service and 20.3% (95%CI 18.9;21.8) had at least one consultation with a specialist physician after diagnosis of COVID-19 (Table 1).

Table 1 presents the prevalence of use of each of the investigated specialist services, in the following decreasing order: cardiologist (13.% 95%CI 12.5;15.0); pulmonologist (7.3% 95%CI 6.4;8.3); psychologist (6.4% 95%CI 5.5;7.3); psychiatrist (4.0% 95%CI 2.9;4.1); physiotherapist (3.4% 95%CI 2.8;4.2); neurologist (3.3% 95%CI 2.7;4.0), and speech therapist (0.6% 95%CI 0.4;1.0). Prevalence of health service use was higher for those with health insurance when compared to those without, with differences of up to 13 p.p (12.8 p.p for specialist services, 12.3 p.p for specialists doctors). Regarding income, the higher income categories had higher prevalence in general services when compared with the poorer income categories, with emphasis on the differences for specialist physicians, specialized services and cardiologists (Table 1).

**Table 1:** Prevalence of health services use according to health insurance and income following COVID-19 in adults and elderly people in the city of Rio Grande. Rio Grande do Sul. Brazil. 2021 (n = 2.919)

Variable	General	Health Insurance		Income			
		No	Yes	Group 1	Group 2	Group 3	Group 4
Specialist doctors^b^	20.3 (18.9;21.8)	13.9 (12.1;15.8)	26.2 (24.0;28.4)	17.4 (14.7;20.5)	18.6(16.3;21.2)	21.6(18.5;25.1)	31.6 (26.5;37.2)
Specialized services^c^	24.7 (23.2; 6.3)	18.0 (16.0;20.1)	30.8 (28.5;33.1)	24.1 (21.0;27.5)	21.8(19.3;24.5)	25.6(22.2;29.2)	37.1 (31.6;42.8)
Pulmonologist	7.3(6.4;8.3)	4.2 (3.2;5.4)	10.1 (8.7;11.8)	6.3(4.7;8.4)	5.6 (4.4;7.2)	7.3 (5.5;9.7)	15.6 (11.9;20.3)
Cardiologist	13.7 (12.5;15.0)	9.4 (8.0;11.1)	17.6 (15.7;19.6)	10.3 (8.2;12.9)	12.5(10.5;14.7)	14.3(11.7;17.3)	22.6 (18.1;27.8)
Neurologist	3.3 (2.7;4.0)	2.6 (1.9;3.6)	3.8 (3.0;4.9)	3.7( 2.5;5.5)	3.1 (2.2;4.4)	2.5 (1.5;4.1)	4.5 (2.6;7.6)
Psychiatrist	4.0 (2.9;4.1)	3.4 (2.5;4.5)	4.5 (3.5;5.6)	4.9 (3.5;6.9)	3.8 (2.9;5.2)	4.0 (2.7;5.9)	4.5 (2.6;7.6)
Physiotherapist	3.4 (2.8;4.2)	2.3(1.7;3.3)	4.3 (3.4;5.5)	3.0 (1.9;4.6)	2.7(1.9;3.9)	3.3 (2.1;5.1)	6.9 (4.5;10.5)
Psychologist	6.4 (5.5;7.3)	6.0 (4.8;7.4)	6.8 (5.6;8.2)	9.5 (7.5;12.0)	5.0 (3.8;6.6)	5.8 (4.2;8.0)	8.7 (5.9;12.6)
Speech therapist	0.6 (0.4;1.0)	0.5 (0.2;0.10)	0.7 (0.4;1.3)	0.8 (0.3;1.8)	0.6 (0.3;1.3)	0.5 (0.2;1.5)	0.3 (0.04;2.5)

^b^ Pulmonologist, cardiologist, psychiatrist, neurologist

^c^ Neurologist, pulmonologist, cardiologist, psychiatrist, psychologist, physiotherapist, speech therapist

In the adjusted analysis, we found that individuals with health insurance had a higher probability of using specialized services, such as pulmonologist (RP = 2.27 95%CI 1.68;3.06), cardiologist (RP = 1. 87 95%CI 1.53;2.29), specialist doctors (RP 1.86 95%CI 1.58;2.19), physiotherapist (RP = 1.83 95%CI 1.16;2.89), neurologist (RP = 1.70 95%CI 1.10;2.63) and specialist services (RP = 1.67 95%CI 1.58;2.19) (Table 2). As for income, the probability of using a physiotherapist, cardiologist, pulmonologist, specialist doctors and specialized services was up to three times higher among the richest compared to the poorest (Table 2).

**Table 2:** Adjusted analysis of health service use variables according to health insurance and income during and after COVID-19 in adults and elderly people in the city of Rio Grande. Rio Grande do Sul. Brazil. 2021 (n = 2.919)

Variable	Health Insurance	Income		
	Yes	Group 2	Group 3	Group 4
Specialists doctors ^b^	**1.86 (1.58;2.19)**	1.04 (0.84; 1.29)	**1.32 (1.04; 1.68)**	**1.96 (1.50; 2.55)**
Specialized services^c^	**1.67 (1.44;1.92)**	0.90 (0.75; 1.08)	1.13 (0.92; 1.38)	**1.64 (1.31; 2.06)**
Pulmonologist	**2.27 (1.68;3.06)**	0.84 (0.57; 1.25)	1.07 (0.68; 1.68)	**2.17 (1.37; 3.44)**
Cardiologist	**1.87 (1.53;2.29)**	1.09 (0.82; 1.45)	**1.47 (1.07; 2.01)**	**2.46 (1.76; 3.45)**
Neurologist	**1.70 (1.10;2.63)**	0.72 (0.42 1.25)	0.75 (0.37 1.50)	1.51 (0.71 3.19)
Psychiatrist	1.42 (0.96;2.09)	0.90 (0.56; 1.44)	1.03 (0.58; 1.81)	1.18 (0.59; 2.38)
Physiotherapist	**1.83 (1.16;2.89)**	0.95 (0.51; 1.76)	1.48 (0.74; 2.94)	**3.13 (1.52; 6.42)**
Psychologist	1.18 (0.87;1.59)	0.61 (0.42; 0.87)	0.74 (0.48; 1.15)	1.13 (0.69; 1.85)
Speech therapist	1.12(0.43;2.96)	0.75 (0.23;2.46)	0.46 (0.12;1.74)	0.25 (0.02;2.57)

Adjusted for: sex, age, skin color, education, marital status and economic class

^b^ Pulmonologist, cardiologist, psychiatrist, neurologist

^c^ Neurologist, pulmonologist, cardiologist, psychiatrist, psychologist, physiotherapist, speech therapist

Regarding the magnitude of socioeconomic inequalities as to income, the health services use such as neurologist, cardiologist, specialists doctors and pulmonologist, respectively presented absolute differences between the prevalence of the first and fourth income groups ranging from 5.34 to 85.62 p.p., while the psychologists use presented absolute differences of -4.28 p.p. represented by IBS. Similarly, relative inequalities (CIX) were significantly higher for pulmonologist, cardiologist and medical inpatient services and lower for psychologist services (Table 3).

**Table 3:** Concentration Index (CIX) and Slope Index of Inequality (SII) of health service use variables according to income following COVID-19 in adults and elderly people in the city of Rio Grande. Rio Grande do Sul. Brazil. 2021 (n = 2.919)

Year	Concentration Index (CIX)	95%CI	Slope Index of Inequality (SII)	95%CI
Specialists doctors ^b^	**6.42**	**2.28; 10.5**	**8.76**	**3.53; 13.9**
Specialized services^c^	4.21	0.48; 7.94	6.25	0.56; 11.9
Pulmonologist	**13.6**	**6.03; 21.2**	**5.34**	**1.78; 8.89**
Cardiologist	**11.1**	**5.92; 16.3**	**9.46**	**4.95; 13.9**
Neurologist	-1.79	-13.7;10.1	**85.62**	**22.24; 149.00**
Psychiatrist	-6.13	-16.1; 3.87	-2.25	-4.82; 0.31
Physiotherapist	9.93	-1.85; 21.7	2.36	-0.14; 4.87
Psychologist	**-11.8**	**-20.30; -3.40**	**-4.28**	**-7.72; -0.83**
Speech therapist	-1.26	-3.00;2.8	-0.13	-1.24;0.97

^b^ Pulmonologist, cardiologist, psychiatrist

^c^ Neurologist, pulmonologist, cardiologist, psychiatrist, psychologist, physiotherapist, speech therapist

The magnitude of socioeconomic inequalities regarding health insurance showed absolute differences ranging from 12.8 to 24.6 p.p. for specialized services, medical specialists, cardiologists, and pulmonologists, respectively. Relative inequalities (CIX) were significantly higher for pulmonologists, medical specialists, cardiologists and specialized services, respectively (Table 4).

**Table 4:** Concentration Index (CIX) and Slope Index of Inequality (SII) of health service use variables according to health insurance following COVID-19 in adults and elderly people in the city of Rio Grande. Rio Grande do Sul. Brazil. 2021 (n = 2.919)

Ano	Concentration Index (CIX)	95%CI	Slope Index of Inequality (SII)	95%CI
Medical specialists services^b^	**15.2**	**11.2;19.3**	**24.6**	**18.7; 30.5**
Specialized services^c^	**11.8**	**8.2;15.7**	**25.5**	**19.4; 31.8**
Pulmonologist	**17.6**	**10.7; 24.5**	**12.8**	**8.4; 17.3**
Cardiologist	**13.8**	**8.7;18.9**	**16.6**	**11.3;21.9**
Neurologist	10.5	-0.69;21.8	2.4	-3.6;5.1
Psychiatrist	4.8	-5.0; 14.6	2.1	-0.8;5.1
Physiotherapist	17.1	6.5; 27.7	4.5	1.4;7.6
Psychologist	1.8	-6.5; 10.1	1.6	-0.2;5.1
Speech therapist	0.5	-22.9;23.9	0.42	-0.79;1.62

^b^ Pulmonologist, cardiologist, psychiatrist

^c^ Neurologist, pulmonologist, cardiologist, psychiatrist, psychologist, physiotherapist, speech therapist

## Discussion

This study identified socioeconomic inequities in the specialized services use in individuals following COVID-19, showing that individuals who have health insurance and who belong to higher income categories are more likely to use specialized services compared to those without health insurance and belonging to lower income categories.

Data from several health systems around the world point out that health inequities are enhanced by existing socioeconomic inequalities [28–30]. Such disparities are also evidenced when it comes to access to specialized services, whether in countries with or without a public health system. In a review study on access to dental services in France demonstrated that individuals in a situation of socioeconomic vulnerability demonstrate greater barriers that hinder or even prevent the receipt of the assistance needed [28, 31].

Neurology services have significant disparities in access and that have been enhanced as a result of the COVID-19 pandemic around the world, socioeconomic issues and structural racism are the main factors preventing equitable access to quality health care and services [29]. In Canada, although the health care system has evolved in recent years, there are still major challenges regarding access to medical specialists, specialized and elective surgeries, as well as a still fragmented and poorly coordinated care logic [30].

Brazilian studies show that the demand for cardiology services during the pandemic experienced a 90% drop; as was the case of a cardiology outpatient clinic compared to the year 2019 [32]. With regard to mental health in a non-pandemic context, a Brazilian study by WANG and collaborators (2017) indicated that only 10% of respondents used a mental health service in the 12 months prior to their health assessment, thus indicating that people with psychiatric problems did not have their treatment performed adequately [33]. Similarly, a Brazilian study compared the speech therapy services use by individuals in public and private settings, finding that people with health insurance (20%) used more of these services when compared to people without health insurance (9%) [34]. Finally, a national study showed that prevalence of use of physiotherapy services was 33% and its use was related to individuals with higher income [35].

There are historical and political factors that can explain the bottleneck for specialized services such as the hegemony of neoliberalism in Brazil, social medicalization, model focused on biomedical care and fragmentation of care. Although the SUS is a system that values universality, equity and integrality in health care and offers quality services to the Brazilian population, still the aspects concerning social inequalities, underfunding and inefficient public-private collaboration threaten its sustainability and guarantee of equal access to all levels of healthcare [5]. If the health policy were really effective and specialized SUS services were well organized, few people would pay health insurance in order to have access to specialized services, which would impact private medicine in Brazil [36, 37].

In our study, individuals who had health insurance were more likely to use specialized health services. Generally, individuals who have better socioeconomic conditions are more likely to have health insurance [38–40], and those who have it are more likely to use health services [41]. The literature also informs that not having health insurance is a factor associated with poorer access to health services [21, 42, 43]. However, it should be noted that about 70% of the population in Brazil depends solely on the SUS, especially individuals of low socioeconomic status.

In addition, in our study there was pro-rich inequality in relation to having health insurance and use of specialized physicians, specialized services, pulmonologists, cardiologists, neurologists and physiotherapists. This data is in line with other studies that show inequalities with regard to restrictions in accessing essential health services [44]. These results have indirect relationships with the Brazilian scenario during the COVID-19 pandemic, such as increased food insecurity in Brazil [45] increased vulnerability, poverty, hunger, unemployment and reduced public health funding further exacerbating existing health inequalities in Brazil [46, 47].

Studies can be found in the literature in which use of health services according to family income classes is unequal and pro-rich in various health services. In fact, specialized services demand a high financial cost, given their complexity and technological density [3, 17]. It is worth noting that when these services are offered by the SUS, in many Brazilian municipalities and regions this is the result of a public-private agreement (for-profit or non-profit and philanthropic), but availability is determined according to market interests and not based on the health needs of the population [48]. In this sense, individuals who depend solely on the SUS end up being put on long waiting lists for care and/or procedures, while those who have higher incomes, and therefore greater purchasing power and high capacity to access these services through private means, are able to meet their needs more immediately [21, 37, 48].

COVID-19 was initially presented as a democratic disease. However, studies show inequalities in the incidence and mortality from COVID-19 in brazil, showing higher mortality among brown, black and indigenous people in most regions of Brazil [49] and higher incidence in the north and northeast regions [50]. This shows that coping conditions were unequal for various population groups and regions in the country, which may have influenced the health services use after covid-19 [51].

The inequalities in specialized services access presented and discussed in this study are related to and intensified by the socioeconomic and political scenario in which the country is immersed. If on the one hand all the achievements in relation to the creation of the SUS, its health care network and programs have favored the supply of and access to health services by the population [3], on the other hand the fiscal austerity measures [52] with health budget freezing through the *Previne* Brazil Program and Technical Note No. 3/2020, which institute new funding rules for Primary Health Care and repeal the federal funding of the Family Health Extended Core and Basic Care (NASF-AB) teams, respectively [53, 54], configure a process of discontinuity and weakening of public health policies. Such movements are articulated in favor of the dismantling of the SUS, and it can be seen that the existing and persistent social inequalities in Brazil are potentiated by the neoliberalist logic, in which social policy can no longer make the right of individuals effective, but rather, contributes to exacerbating inequalities and defining access to services based on each person’s purchasing power [55, 56].

It is worth mentioning that this study is not exempt from limitations. Among them, we highlight the possibility of memory bias and/or residual confusion in the questions about the health services use, since participants might not remember these details accurately. Finally, because we did not interview asymptomatic individuals, we cannot conclude whether or not these individuals required greater use of health services following infection. Despite the limitations mentioned above, this study has potential because it deals with a theme that has been little investigated in the scientific literature. Moreover, by performing specific analyses of inequalities using the SII and CIX indicators that take into consideration not only extreme groups, but the entire distribution of data, it demonstrates a methodological quality/adequacy capable of identifying the specificities existing on the access to specialized health services according to health insurance and income. Such measures are essential for identifying existing disparities and contributing to a better targeting of public policies, targeting the population in situations of social and health vulnerability.

## Conclusion

We therefore conclude that socioeconomic inequalities exist in the specialized services use by individuals following COVID-19 in the far south of Brazil. We believe that the results of this study can be useful in signaling the need to reduce the difficulty in accessing and using specialized services. One of the possibilities for reducing the difficulty in accessing and using specialized services is to consider technological alternatives in an articulated network (referral and counter-referral) such as telehealth, especially in places far away from large health centers. Moreover, it is also important to emphasize the need to increase training and qualification of health professionals, and financial incentives for these professionals to remain in very remote areas. The SUS has a relevance as a universal system and a policy that guarantees the right to health for all individuals, and, facing a scenario of underfunding and dismantling, it is necessary to join efforts to defend it and to ensure that the social and health policy is in fact effective.

The study was carried out with financial support from FAPERGS - Research Support Foundation of Rio Grande do Sul, Brazil grant number 21/2551-0000107-0 Research Program for the SUS: shared management in health - PPSUS).

## Data Availability

Datasets used and/or analyzed during the current study are available from the corresponding author upon reasonable request.

## List of Abbreviations

SUS: Unified Health System
RT-PCR: Reverse transcriptase reaction- Polymerase chain reaction
NASF-AB: Family Health Extended Core and Basic Care
SII: Slope Index of Inequality
CIX: Concentration Index
